# Hematological indices in psoriatic enthesopathy: relation to clinical and ultrasound evaluation

**DOI:** 10.1007/s10067-024-06951-2

**Published:** 2024-04-08

**Authors:** Arwa S. Amer, Ahmed Y. Al Shambaky, Seham G. Ameen, Amira Khalil Sobih

**Affiliations:** 1 Rheumatology, Rehabilitation, and Physical Medicine, Faculty of Medicine, Benha University, Fareed Nada Street, Benha, Qalubiya Governorate 13511 Arab Republic of Egypt; 2 Clinical and Chemical Pathology, Faculty of Medicine, Benha University, Fareed Nada Street, Benha, Qalubiya Governorate 13511 Arab Republic of Egypt; 3 Faculty of Medicine, Benha University, Fareed Nada Street, Benha, Qalubiya Governorate 13511 Arab Republic of Egypt

**Keywords:** Enthesopathy, LEI, MPV, Musculoskeletal ultrasound, NLR, Psoriasis, RDW

## Abstract

**Background:**

Enthesopathy is considered a crucial aspect of assessment and outcome in psoriatic arthritis (PsA). Musculoskeletal ultrasound (MSUS) is a critical tool for accurately detecting enthesitis. Recent research focuses on identifying simple biomarkers for detecting and monitoring psoriatic enthesopathy. Red cell distribution width (RDW), mean platelet volume (MPV), and neutrophil/lymphocyte ratio (NLR) are components of a complete blood count (CBC) and are reliable bio-inflammatory markers in various rheumatic diseases.

**Aim of work:**

To measure MPV, RDW, and NLR in psoriatic enthesopathy and determine their relationship to disease activity and MSUS findings.

**Patients and methods:**

This study focused on 30 people with psoriatic arthritis (PsA) as per CASPAR criteria, along with 20 control subjects. Enthesopathy was evaluated clinically using the Leeds Enthesitis Index (LEI). The modified Disease Activity Index of Psoriatic Arthritis (DAPSA28) was calculated, and RDW, MPV, NLR, CRP, and ESR were measured. Each enthesis in LEI was radiologically assessed using plain radiography and MSUS according to OMERACT definitions.

**Results:**

There was a significant relationship between clinical tenderness, the presence of enthesophytes on plain radiography, and MSUS findings at entheses sites (*p* < 0.001 for each). Psoriatic patients had higher levels of RDW and MPV (*p* < 0.001 and 0.01, respectively) than controls, with no significant differences in NLR (*p* = 0.189) between the two groups. RDW and MPV levels were positively correlated with the DAPSA28 score.

**Conclusion:**

Monitoring PsA disease activity can be improved by considering RDW and MPV as reliable indicators and using them to screen for psoriatic enthesopathy with MSUS indices.

**Table:** 

**Key points**
• *Clinically identifying enthesitis in patients with PsA can be challenging. Imaging MSUS indices hold promise for objective analysis, but there is no consensus on which indices to use in clinical trials and daily practice.* • *Patients with psoriatic enthesopathy have higher RDW and MPV levels, which are positively correlated with DAPSA28 score.* • *RDW and MPV can be considered in the turn of improved screening of psoriatic enthesopathy with MSUS scores.*

## Introduction

Enthesitis refers to the inflammation that occurs at the point where a tendon, ligament or capsule attaches to a nearby bone [1, 2]. This condition is prevalent in 35–50% of patients with psoriatic arthritis (PsA) [3, 4]. It is believed to be a triggering mechanism in individuals with spondyloarthropathy, a group of diseases that includes PsA [5, 6]. Recent studies have linked enthesitis to radiographic damage in both peripheral and axial joints of PsA patients [4]. Overall, enthesitis is becoming an increasingly important marker that needs to be evaluated and monitored more closely.

The effectiveness and precision of clinical evaluations for examining entheses fall below acceptable standards, and imaging methods hold promise for their objective analysis [7]. One such technique is MSUS, which is safe and cost-effective [8]. In the assessment of enthesitis, ultrasound imaging can reveal a variety of abnormalities, including bursitis, formation of enthesophytes, disruption of the typical fibrillar structure, diminished echogenicity, swelling in the subcutaneous tissues, enlargement of the enthesis, and bone erosions [9]. Additionally, Doppler imaging is capable of identifying increased blood flow within synovial tendon sheaths, as well as in bursae and entheses [10].

Recent research is currently focused on discovering simple biomarkers that play a key role in the screening and tracking of psoriatic enthesopathy. These biomarkers can enhance diagnostic confidence, serve as a prognostic indicator, facilitate clinicians in selecting treatment options, and assist in tracking the progression of the disease. Complete blood cell parameters (CBC) have become a valuable and cost-effective tool for assessing systemic inflammation in various rheumatic diseases [11]. Platelets play a crucial role in immune responses and inflammatory reactions as they gather at the site of damage and adhere to white blood cells. This process triggers the release of cytokines and chemokines which attract neutrophils, lymphocytes, and monocytes to the damaged area, enhancing the inflammation [12]. They can also trigger inflammation by releasing inflammatory prostaglandins and increase vascular permeability [11, 13]. The mean platelet volume (MPV) is a commonly used metric to determine the size of platelets and can potentially indicate disease activity. MPV is included in routine CBC analysis, making it a cost-effective and easy method to measure platelet activation in daily practice. The red cell distribution width (RDW) is a recently described inflammatory biomarker that is mainly attributed to the ability of RDWs to reliably reflect an increase in the levels of circulating cytokines such as tumor necrosis factor-alpha (TNF-alpha), interleukin-6 (IL-6), and hepcidin [14]. Chronic inflammation has an impact on the development and lifespan of red blood cells. It also affects the sensitivity of erythropoietin, contributing to an increase in RDW [15]. Moreover, the value of the neutrophil-to-lymphocyte ratio (NLR) as an inflammatory marker increases due to neutrophils’ ability to produce TNF-α in the site of inflammation, which in turn recruits and stimulates B and T lymphocytes [11]. The purpose of this research was to evaluate MPV, RDW, and NLR in individuals with psoriatic enthesopathy, aiming to correlate these parameters with disease activity and MSUS evaluation.

## Patients and methods

### Study design

This case-control research included 50 subjects, with 20 serving as controls and 30 PsA patients who met CASPAR criteria [16]. Recruitment occurred at Benha University’s departments of Rheumatology, Rehabilitation, and Physical Medicine from October 2021 to October 2022, targeting individuals aged over 18. Skin and nail psoriatic changes were diagnosed by a consultant dermatologist in the hospital. Exclusion criteria were as follows: patients with seronegative spondyloarthropathies other than PsA, crystal-induced arthritis, osteoarthritis (OA), and metabolic and endocrinal disorders and patients who have undergone a corticosteroid injection at scanned entheses or have active skin inflammation other than psoriasis as well as who previously treated with retinoids. The study was conducted per the Declaration of Helsinki and approved by the Ethics Committee of Benha University’s Faculty of Medicine (Ms. 23.9.2021). Each patient provided informed consent before the study.

### Clinical and disease activity assessment

A senior rheumatologist conducted a thorough medical history and clinical examination for all the patients, independent of imaging studies. The Leeds Enthesitis Index (LEI) was employed to gauge entheses tenderness by exerting approximately 4 kg/cm^2^ of pressure at six specific locations: Achilles tendons insertion, medial femoral condyles, and lateral elbow epicondyles. Tenderness was scored from 0 (no tenderness) to 1 (presence of tenderness) [17, 18]. Furthermore, the disease’s activity was determined using the modified Disease Activity Score for 28 joints (DAPSA28), which incorporates tender and swollen joint counts (TJCs and SJCs), C-reactive protein (CRP) levels, patient self-reports of disease activity, and pain assessments. This score ranges from 0 to 28, with a score of 4 or lower indicating remission, over 4 to 14 suggesting low disease activity, over 14 to 28 signifying moderate disease activity, and above 28 indicating high disease activity [19].

### Laboratory investigations

Laboratory investigations were performed, including CBC, MPV, NLR, RDW, ESR, CRP, RF, FBS, serum creatinine, urea, uric acid, ALT, and AST.

### Radiological investigations

1. Plain imaging radiography was performed on the sacroiliac joints, hands, elbows, knees, feet, and ankles.
2. MSUS evaluation of entheses:

The musculoskeletal ultrasound scans were conducted by a skilled rheumatologist who was blinded to the clinical examination. The ultrasound scans were performed on the same day of clinical examination using the GE LOGIQ P9 ultrasound machine equipped with an 8–13 MHz multi-frequency linear transducer. The assessment was made at each of the Leeds enthesis sites (Achilles tendons insertion, medial femoral condyles, and lateral elbow epicondyles). The patient’s joints were positioned according to EULAR recommendations; tendons were stretched to avoid anisotropy and were unflexed to assess PD signals [20]. The tendo-Achilles insertion was examined with the patient lying prone, feet extending beyond the couch, and ankles in a neutral stance. The MCL insertion into the femur was examined with the patient lying supine, knees slightly bent at 20–30 degrees. The CEO of the humerus was evaluated with elbows slightly bent, hands on knees, and forearms rotated inward. Imaging was performed along both longitudinal and transverse sections. Each enthesis was scanned in gray scale to detect morpho-structural changes, and subsequently, with power Doppler (PD) to detect abnormal blood flow, as defined by the Outcome Measures in Rheumatology (OMERACT) Ultrasound Task Force [21, 22]. Grayscale ultrasound identified features such as erosions (step-down cortical breaks visible in two dimensions, exceeding 2 mm in diameter), enthesophytes (bony projections at the tendon-bone junction), bursitis (clearly outlined hypoechoic regions at bursal locations), entheseal thickening (uneven thickness compared to the opposite side), and soft tissue edema (fluid accumulation at the enthesis edge). Power Doppler ultrasound was employed to detect neovascularization near the entheseal insertion. Findings were scored if seen in both planes as either absent (0) or present (1). These ultrasound observations were aggregated into two categories: an inflammation score summing vascularization, edema, bursitis, and entheseal thickening (ranging from 0 to 4), and a damage score comprising erosion and enthesophyte assessments (ranging from 0 to 2).

### Statistical analysis

The analysis of data within this research utilized IBM SPSS Statistics software, version 25.0 (IBM Corp., Armonk, NY, USA, released in 2017). A variety of statistical assessments were employed to evaluate the significant differences among the groups under investigation. These assessments included Student’s *t*-test, chi-square test, one-way ANOVA, Mann–Whitney *U* test, Kruskal–Wallis test, and Fisher’s exact test. The ROC curve analysis was implemented to determine the diagnostic accuracy of hematological markers in differentiating between the study groups. A *p*-value of 0.05 or less was considered statistically significant.

The sample size was calculated by Stata Corp. 2021 (Stata Statistical Software: Release 17) (College Station, TX: Stata Corp LLC). In a case–control study, using a *t*-test model, the difference between two independent means, the expected effect size of 0.9, the required minimal sample size is 20 subjects in each group, using α error of 5% and a power of 80%. The number of cases was increased to 30 patients to increase the power of the study.

## Results

In a study of 30 individuals with psoriatic enthesopathy, 70% were female and 30% male, with an average age of 42.7 years. The study also included 20 healthy control subjects. Statistical analysis revealed no significant differences in age or gender between patients and controls (*p* = 0.792 and *p* = 0.311, respectively). The median disease duration among patients was 12.5 years ranging from 2.0 to 50.0 years, and 36.7% reported a family history of psoriasis. The median DAPSA28 score was 18.05, with a range from 1.40 to 33.80. Patient disease activity was classified as follows: high activity in 2 (6.7%) patients, moderate in 19 (63.3%), low in 6 (20.0%), and remission in 3 (10.0%). Details on the clinical characteristics and treatment approaches for the patient group are summarized in Table 1.

**Table 1:** Clinical findings and treatment approaches of patients’ group

	Psoriatic enthesopathy *N* = 30	
NTJ (mean ± SE)	4.07 ± 0.59	
NSJ (mean ± SE)	1.47 ± 0.42	
Skin psoriasis	20	66.7%
Nail psoriasis	9	30.0%
Entheses tenderness	No	**%**
Rt CEO	15	50.0%
Lt CEO	15	50.0%
Rt MCL	26	86.7%
Lt MCL	23	76.7%
Rt Ach	13	43.3%
Lt Ach	17	56.7%
Medications	No	**%**
MTX	6	20.0
Leflunomide	14	46.7
Cyclosporine	1	3.3
Sulfasalazine	2	6.7
IL17 inhibitor	13	43.3
Etanercept	4	13.3
Adalimumab	4	13.3
Golimumab	1	3.3

*NTJ* number of tender joints, *NSJ* number of swollen joints, *CEO* common extensor origin, *MCL* medial collateral ligament of the knee, *Ach* Achilles tendon, *MTX* methotrexate

**Fig. 1:**
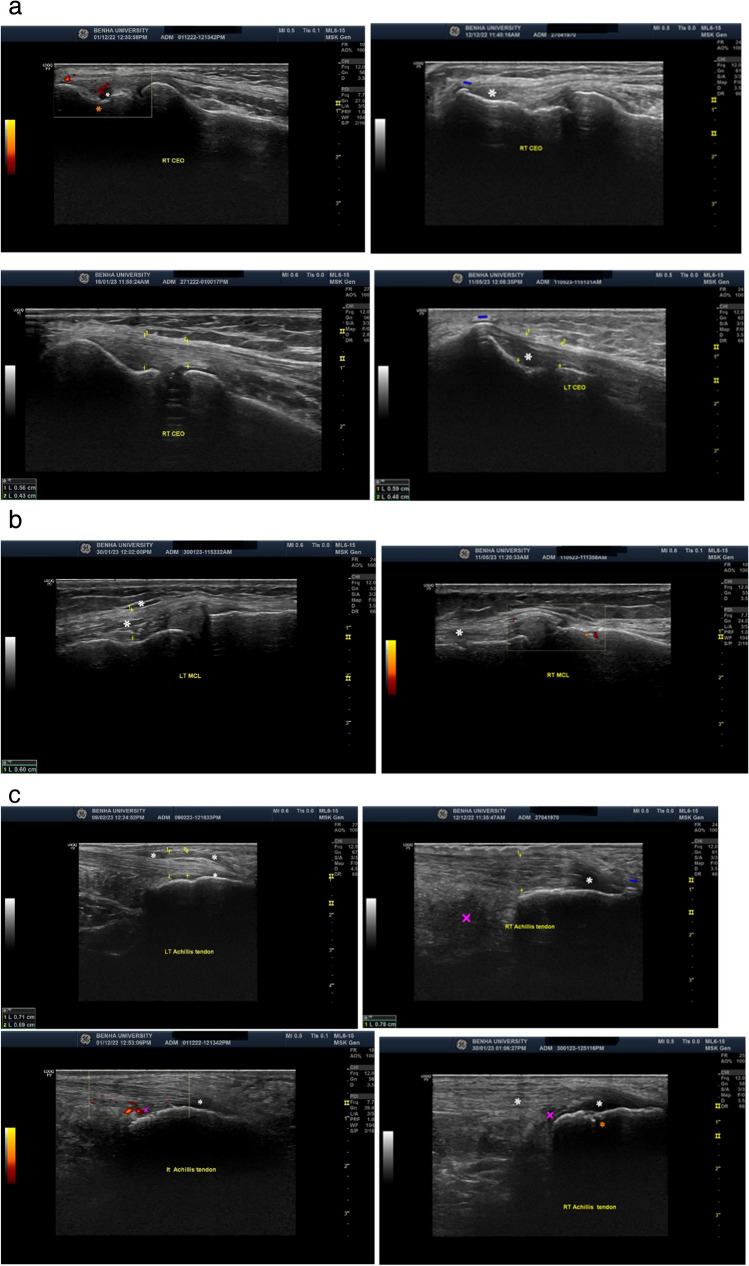
MSUS findings at sites of LEI entheses. **a** CEO enthesopathy in the form of increased thickness, soft tissue edema, presence of enthesophyte, erosion, and active Doppler signals. **b** MCL enthesopathy in the form of increased thickness, soft tissue edema, and erosion with an active Doppler signal. **c** Achilles tendon enthesopathy in the form of increased thickness, soft tissue edema, presence of enthesophyte, active Doppler bursitis, and erosion. White asterisk (*): hypoechoic area and soft tissue edema; orange asterisk (*): erosion; blue MDASH (–): enthesophyte; violet cross (×): bursa; yellow plus sign (+): thickness

During clinical and radiological evaluation of entheses, a significant association was observed between clinical tenderness, enthesophyte presence on plain radiography, and MSUS findings at CEO, MCL, and tendo-Achilles insertion (*p* < 0.001 for each). Also, a statistically significant association was found between the presence of enthesophytes on plain radiography at lateral elbow epicondyles, both medial femoral condyles and the MSUS damage score (*p* < 0.05 each). Table 2 presents the damage and inflammatory MSUS scores of the patients’ group and their plain radiography and MSUS findings, as shown in Fig. 1a, b, and c.

**Table 2:** The damage and inflammatory scores of the patients’ group and their MSUS and plain X-ray findings for LEI

	Psoriatic enthesopathy *N* = 30			
	Right		Left	
Plain X-ray	No	%	No	%
Enthesophyte at CEO	6	20.0	3	10.0
Enthesophyte at Ach	17	56.7	8	26.7
Enthesophyte at MCL	8	26.7	5	16.7
MSUS findings	No	**%**	No	**%**
Erosion				
CEO	5	16.7	6	20.0
MCL	2	6.7	3	10.0
Ach	1	3.3	2	6.7
Enthesophyte				
CEO	6	20.0	9	30.0
MCL	6	20.0	8	26.7
Ach	16	53.3	20	66.7
Bursitis				
CEO	2	6.7	0	0.0
MCL	0	0.0	2	6.7
Ach	13	43.3	16	53.3
Entheseal thickening				
CEO	6	20.0	2	6.7
MCL	15	50.0	18	60.0
Ach	8	26.7	11	36.7
Soft tissue edema				
CEO	13	43.3	18	60.0
MCL	17	56.7	21	70.0
Ach	13	43.3	20	66.7
Doppler signal				
CEO	7	23.3	11	36.7
MCL	6	20.0	5	16.7
Ach	4	13.3	7	23.3
MSUS scores	Median	Min.–Max	Median	Min.–Max
Inflammation score				
CEO	1.0	0.0–3.0	1.0	0.0–2.0
MCL	1.50	0.0–3.0	2.0	0.0–3.0
Ach	1.0	0.0–3.0	2.0	0.0–4.0
Damage score				
CEO	0.0	0.0–2.0	0.0	0.0–2.0
MCL	0.0	0.0–1.0	0.0	0.0–2.0
Ach	1.0	0.0–1.0	1.0	0.0–2.0

Inflammation score involves vascularization (positive power Doppler), soft tissue edema, bursitis, and entheseal thickening. Damage score involves erosion and enthesophyte

*CEO* common extensor origin, *MCL* medial collateral ligament of the knee, *Ach* Achilles tendon

Our study showed that the measurements of MPV and RDW were notably elevated in the patient’s group (*p* = 0.001 and *p* = 0.010, respectively), whereas no significant variance was found in the NLR (*p* = 0.189). Additionally, levels of ESR, ALT, CRP, and AST were significantly increased in individuals with psoriatic enthesopathy (*p* < 0.05). Conversely, the concentrations of serum creatinine, urea, and uric acid did not show significant differences (*p* > 0.05).

**Fig. 2:**
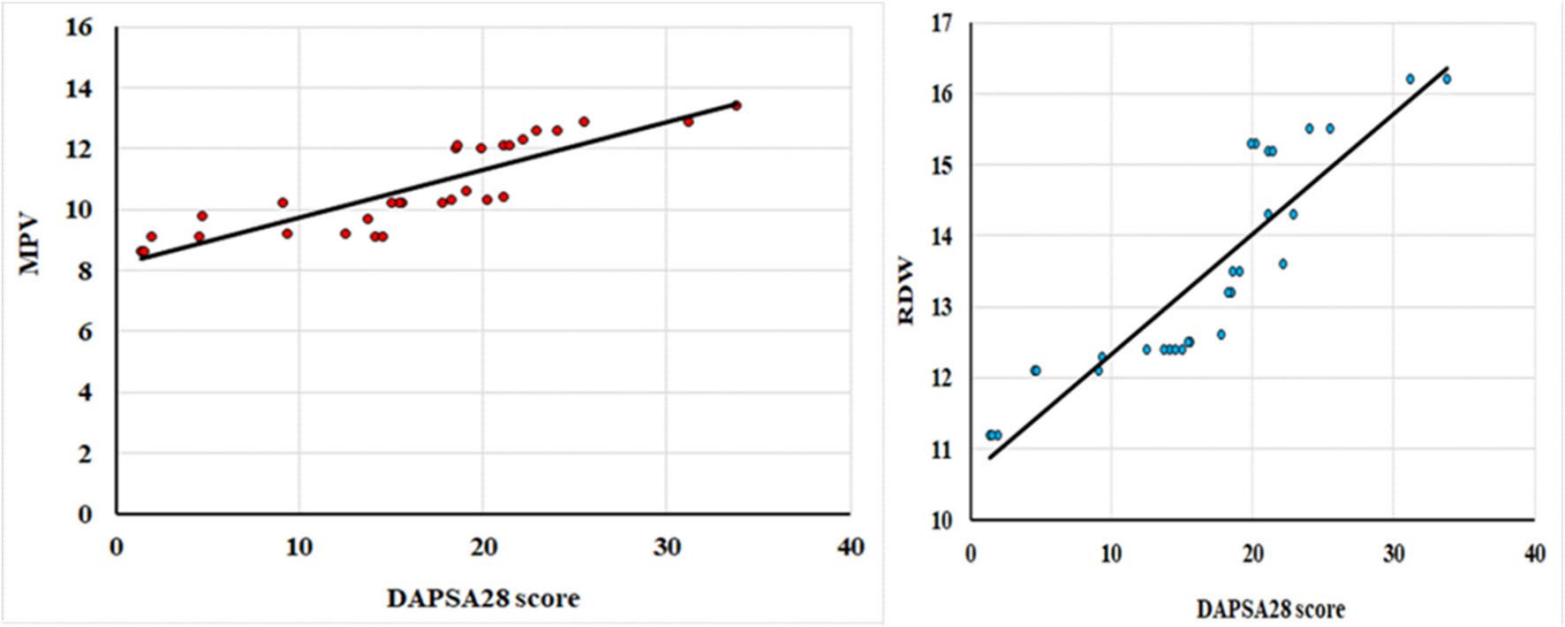
Correlation of MPV and RDW with DAPSA28 score in patients with psoriatic enthesopathy

The study revealed a significant positive association between both MPV and RDW with the number of tender joints (TJ), number of swollen joints (SJ), and the DAPSA28 score as depicted in Fig. 2, each demonstrating strong correlations (*p* < 0.001). Furthermore, MPV was found to correlate significantly with RDW (*p* < 0.001). The NLR was significantly correlated with CRP levels (*p* = 0.003) and the total leukocyte count (TLC) (*p* < 0.001), yet it did not show a significant correlation with the DAPSA28 score. Notably, MPV and RDW values varied significantly across different DAPSA28 categories (*p* < 0.001), with higher levels observed in cases of moderate and high disease activity compared to those in low activity or remission.

**Fig. 3:**
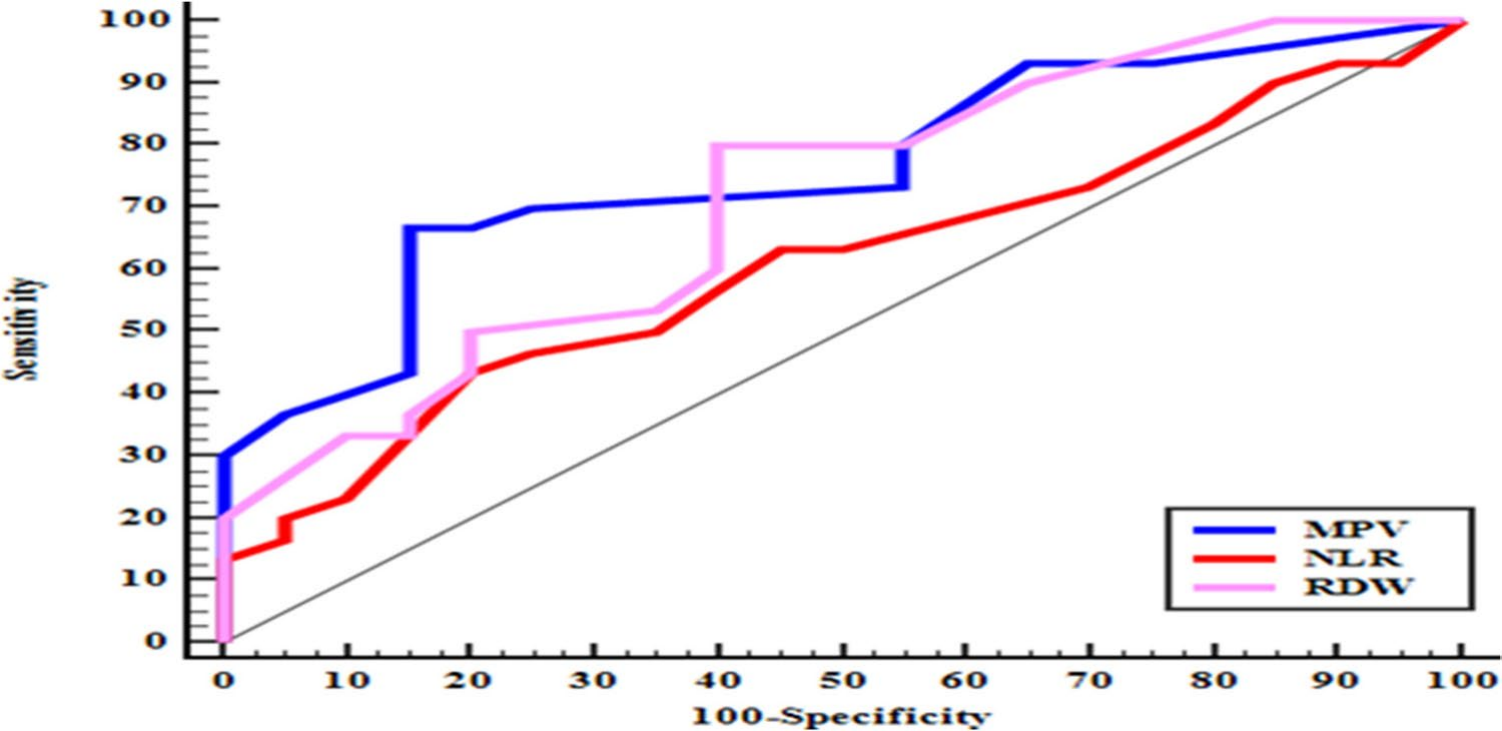
ROC curve for hematological indices to distinguish patients with psoriatic enthesopathy from the control group

The ROC curve analysis of hematological indices was conducted to distinguish between the studied groups. The analysis showed that MPV had a best cutoff value of more than 9.7, with moderate accuracy (area under the curve (AUC) = 0.761), 70% sensitivity, 70% specificity, and 80.77% positive predictive value (PPV). RDW had a best cutoff value of more than 12.2%, with moderate accuracy (AUC = 0.714), 80% sensitivity, 60% specificity, and 75% PPV. On the other hand, NLR had a best cutoff value of more than 2.5%, with low accuracy (AUC = 0.610), sensitivity of 63.33%, specificity of 55%, and PPV of 67.86%, Fig. 3.

## Discussion

Identifying enthesitis in patients with psoriatic arthritis (PsA) can be difficult due to clinical limitations. Therefore, imaging techniques such as ultrasound (US) and magnetic resonance imaging (MRI) have become increasingly important. Different indices have been proposed to assess enthesitis in PsA patients, but there is no consensus on which ones to use in clinical trials or daily practice [23]. Recent research is focusing on the discovery of simple biomarkers that aid in screening of psoriatic enthesopathy which may improve diagnostic and therapeutic confidence. MPV, RDW, and NLR are considered recent potential inflammatory markers associated with several rheumatological diseases [11]. This study aimed to measure MPV, NLR, and RDW in patients with psoriatic enthesopathy, to find out their relations to disease activity and enthesopathy MSUS evaluation. Many researchers have studied the relationship between MPV, RDW, and NLR in psoriatic arthritis (PsA) and psoriasis; however, this is the first study that investigated the hematological indices in psoriatic enthesopathy. Furthermore, the current study used the LEI score, which is the only index developed and validated specifically for psoriatic enthesopathy among MSUS scores.

In the present study, clinical and MSUS evaluation of enthesopathy revealed highly statistically significant associations between clinical tenderness, the presence of enthesophyte on plain radiography, and MSUS inflammatory and damage scores at entheses sites, and this is consistent with Ahmad et al. [24], who showed a notable positive correlation between the occurrence of tenderness and the MSUS inflammatory score. However, there is a difference in the lack of correlation with the damage score, which could be attributed to the relatively shorter study duration of their research (median of 4 years) in comparison to ours (12.5 years). A positive, significant correlation was found between the presence of Achilles tendon tenderness and MSUS inflammatory and damage scores by Ahmed et al. [25], which agrees with our findings. On the other hand, Agache et al. [26] discovered a weak correlation between tenderness and inflammatory or damage scores at the tendo-Achilles entheses, and this could be attributed to the fact that our patients had a higher disease activity (median DAPSA score of 18.05), which is considerably higher than their patients (8.8).

As regards hematological indices, our research identified significant disparities in MPV and RDW levels among the groups under study, aligning with the outcomes of studies by Aboud et al. [27] and Kim et al. [28]. Yet, our findings diverge from those reported by Saleh et al. [29] and Safina et al. [30], who observed no significant differences in MPV levels between their patient and control groups. They explained their different results as psoriasis’s nature is a chronic inflammatory condition that prompts an increased platelet accumulation in the skin, leading to fewer circulating platelets [12, 31].

Conversely, Hammad et al. [32] documented a significant elevation in NLR levels among their study’s patient group versus controls. Our analysis, however, did not reflect a significant variance in NLR levels, a discrepancy possibly due to Hammad’s inclusion solely of patients suffering from psoriasis vulgaris, a particularly severe and acute form of psoriasis [12].

Our research has revealed that both MPV and RDW have a strong positive correlation with the number of tender joints, swollen joints, and DAPSA28 score. This finding is consistent with the results of previous studies by Aboud et al. [27] and Ozisler et al. [33]. They observed that RDW has a positive correlation with the DAPSA28 score, while Mustafa et al. [34] reported that MPV positively correlated with the DAPSA28 score. Additionally, our findings are also supported by Raghavan et al. [35], who reported that MPV is positively correlated with RDW.

Aboud et al. [27] and Bożena et al. [36] have reported that there is no statistically significant difference between NLR levels in PsA patients and DAPSA28 score grades, which is consistent with our study results. However, we discovered that NLR demonstrated a statistically significant correlation with TLC and CRP, which agrees with Aboud et al. [27].

In the context of other laboratory investigations, our research aligns with those reported by AlJohani et al. [37] and Moustafa et al. [34], indicating notably elevated levels of ESR and CRP in patients relative to control subjects. Similarly, we agree with Van et al. [38], who identified a significant increase in ALT levels in the patient group compared to the control group, whereas Wang et al. [39] reported no significant differences in their study. Our research showed that there were no significant differences in the levels of serum creatinine, urea, and uric acid between the groups that we compared. However, Khan et al. [40] found that the patients had significantly higher levels when compared to the controls. Additionally, Moustafa et al. [34] found a significant difference in the level of serum uric acid between the groups they studied. This difference could be attributed to the fact that the present study had fewer cases of skin involvement compared to others. As a result, our study may not have been sufficient enough to induce hyperuricemia.

In our study, we assessed the ability of hematological indices to differentiate psoriatic enthesopathy from the control group using ROC analysis. The best cutoff value for MPV was > 9.7, with a sensitivity of 70%, a specificity of 70%, and a PPV of 80.77%. For RDW, the best cutoff value was > 12.2, with a sensitivity of 80%, a specificity of 60%, and a PPV of 75%. Aboud et al. [27] identified the best RDW cutoff value for disease activity prediction as > 13.2, with a specificity of 100.0%, a sensitivity of 72.50%, and 100.0% PPV. The best MPV cutoff value was > 8.4, with a specificity of 70.0%, sensitivity of 67.50%, and PPV of 81.8%.

It is important to acknowledge that our study has certain limitations. Firstly, the data used for the study was obtained from a single hospital, which may have led to bias in patient selection. Secondly, all cases included in the study were receiving steroids and immunosuppressants, which could have affected the levels of hematological indices. Finally, we did not have access to any information regarding the nutritional status or nutritional elements, such as levels of vitamin B12, folic acid, or iron, and this lack of information may have resulted in changes to CBC parameters.

In conclusion, RDW and MPV are promising biomarkers of PsA disease activity, and they may also serve as indicators of psoriatic enthesopathy with MSUS indices. Prospective further studies are required to investigate such hematological indices in early diagnosis of psoriatic enthesopathy.

## Data Availability

The datasets used and analyzed during the current study are available from the corresponding author upon reasonable request.
